# Cow Milk Fatty Acid and Protein Composition in Different Breeds and Regions in China

**DOI:** 10.3390/molecules29215142

**Published:** 2024-10-30

**Authors:** Yunxia Zou, Yifei Chen, Qingyong Meng, Yachun Wang, Yali Zhang

**Affiliations:** 1 College of Food Science and Nutritional Engineering, China Agricultural University, Beijing 100083, China; zouyx9@163.com (Y.Z.); 15214685253@163.com (Y.C.); 2 College of Biological Science, China Agricultural University, Beijing 100193, China; qymeng@cau.edu.cn; 3 College of Animal Science and Technology, China Agricultural University, Beijing 100193, China

**Keywords:** bovine milk, breeds, fatty acids, protein composition, region

## Abstract

Cow milk is rich in proteins, fats, carbohydrates, and minerals; however, its precise nutrient content varies based on various factors. In the current study, we evaluated the differences in the fatty acid and protein contents of milk and the factors associated with these differences. To achieve this, samples were collected from seven types of cows in different regions. These included samples from three dairy breeds: Chinese Holstein milk from Beijing, China (BH), Chinese Holstein milk (HH) and Jersey milk (JS) from Hebei province, China; and four dairy/meat breeds: Sanhe milk (SH) from Inner Mongolia Autonomous Region, China, Xinjiang brown milk (XH) and Simmental milk (SI) from Xinjiang Uygur Autonomous Region, China, and Shu Xuanhua milk (SX) from Sichuan province, China. Breed significantly affects total fat, fatty acid, and protein contents. Additionally, geographic region significantly affects the contents of different fatty acids, α-lactalbumin, and lactoferrin. JS has the highest total fat and casein contents. XH samples contain significantly higher unsaturated fatty acid content than BH samples and do not differ significantly from JS. Additionally, the low β-lactoglobulin and high lactoferrin contents in XH samples may be favorable for the growth and development of infants. Our results may inform the development of dairy products from different cow breeds and advance the process of accurate breed identification.

## 1. Introduction

Milk is a complex fluid produced by the mammary glands that meets the nutritional needs of newborn mammals during the early critical period of physical development. Milk is also a high-quality source of dietary proteins for humans, predominantly categorized into casein and whey proteins. In particular, cow milk provides essential nutrients, including proteins, lipids, lactose, vitamins, and minerals [1]. Indeed, 95% of dairy products originate from cow milk globally, excluding Mediterranean Basin countries [2]. Due to its unique amino acid composition, cow milk proteins also serve as a source of various bioactive peptides [3]. However, lipids are the major components of milk, with triacylglycerols, including fatty acids of different lengths and saturations, comprising approximately 98% of the lipid composition [4].

Based on their saturation levels, fatty acids are categorized as saturated fatty acids (SFAs; 0 double bonds), monounsaturated fatty acids (MUFAs; 1 double bond), and polyunsaturated fatty acids (PUFAs; ≥2 double bonds, 18–22 carbons). PUFAs with the first double bond on the third carbon atom from the methyl end are n-3 fatty acids, including linolenic acid (C_18_H_30_O_2_), eicosapentaenoic acid (C_20_H_30_O_2_), and docosahexaenoic acid (C_22_H_32_O_2_). Meanwhile, those with the first double bond on the sixth carbon atom are n-6 fatty acids, including linoleic acid (C_18_H_32_O_2_) and arachidonic acid (C_20_H_32_O_2_) [5].

The composition of cow’s milk is influenced by various factors, including season, feed, breed, lactation period, parity, and udder health [6]. Holstein cows are a globally popular dairy breed, with the highest milk production and largest number of cows bred [7]. In fact, more than 85% of the nearly 10.44 million dairy cows raised in China are Chinese Holstein dairy cows, with a small number of Jersey cows [8]. Chinese Holstein cows are bred by crossbreeding local yellow cows with imported Holstein cows [9]. Meanwhile, Jersey cows are among the oldest dairy breeds; their milk contains high levels of fat and protein [10]. Several other dairy/meat cattle breeds, such as Sanhe, Xinjiang brown, Simmental, and Shu Xuanhua cattle, are also bred in China. Sanhe cattle, originating from the Inner Mongolia Autonomous Region of China, have a high tolerance to roughage, high adaptability, disease resistance, and stable genetic properties [11]. They arise from complex crossbreeding of more than ten breeds of Simmental, Zabaikal, and Siberian cattle under the ecological conditions of the steppe [12]. Meanwhile, Xinjiang Brown dairy/meat cattle originate from the successive crossing of female Kazakh cattle with male Swiss Brown, Alatau, and Kostrom cattle and through long-term selection and breeding [13]. They are highly adaptable and resistant to extreme weather conditions and, thus, commonly graze in the northern part of Xinjiang, China [14]. One of China’s largest Simmental cattle farms is also located in Xinjiang. While the original Simmental cattle were imported from Europe, the Ministry of Agriculture considers them the main cow breed in the country due to their high milk production, meat quality, disease resistance, and tolerance for roughage [15]. Meanwhile, the Shu Xuanhua cattle pedigree includes yellow, Simmental, and Holstein cows adapted to the high temperatures and humidity in natural climates with a diet high in roughage and management conditions. They are primarily raised in the agricultural areas of southern China [16].

Current studies on the milk nutrient composition of Chinese dairy cows have focused on Chinese Holstein and Jersey cows. However, few studies have compared the nutrients in Holstein milk with those in milk of other Chinese dairy cow breeds. These dairy/meat cattle are an important source of meat and milk for people living in different regions of China; however, data on the main nutritional components of dairy/meat cattle’s milk are lacking. Moreover, although the fatty acid and protein content of cow’s milk has been reported, these studies have focused on the total nutrient content, lacking a systematic analysis of the specific content breakdown. Additionally, while patterns in milk component changes have been evaluated within a single breed, there is a general lack of comparison among breeds [17,18,19].

The aim of this study is to evaluate the main nutrient compositions of cow milk in different breeds in China. The total fat, 17 fatty acids, casein (CN), β-lactoglobulin (β-Lg), α-lactalbumin (α-La), and lactoferrin (LF) contents are evaluated in the milk of Chinese Holstein cows (from Beijing, China (BH), and Hebei, China (HH)), Jersey cows (JS), Sanhe cattle (SH), Shu Xuanhua cattle (SX), Simmental cattle (SI), and Xinjiang brown cattle (XH). Collectively, the results of this section provide a reference for research on local specialty cattle breeds in China and inform the development of improved breeding strategies. In particular, this information can be applied to concentrate certain active substances in milk to accelerate the development and utilization of Chinese cattle breeds.

## 2. Results

### 2.1. Milk Fat Content per Breed/Region

**Figure 1:**
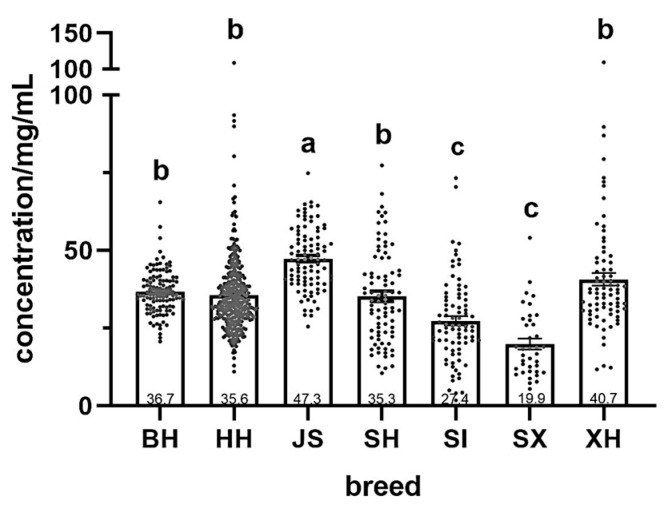
Fat content of milk samples from different cow breeds in different regions in China. BH (*n* = 115), HH (*n* = 348), JS (*n* = 94), SH (*n* = 84), SI (*n* = 82), SX (*n* = 38), XH (*n* = 78). Values with different letters are significantly different at p < 0.05. BH = Beijing Holstein milk, HH = Hebei Holstein milk, JS = Jersey milk, SH = Sanhe milk, SI = Simmental milk, SX = Shu Xuanhua milk, XH = Xinjiang brown milk. Each dot indicates the milk fat concentration for each cow.

Figure 1 presents the fat content of different milk samples. JS samples had the highest fat content (47.3 ± 1.0 mg/mL), significantly higher than those from other breeds and 1.3-fold higher than BH. SX had the lowest fat content (19.9 ± 1.7 mg/mL), significantly lower than that of BH, HH, JS, SH, and XH. No significant differences were observed between the fat contents of BH, HH, SH, or XH samples. Importantly, the fat content did not differ between the two Holstein milk samples from different regions (BH vs. HH). Meanwhile, SH and XH milk samples contained significantly higher fat content than SI. Overall, the total fat content did not differ among the milk samples from the four dairy/meat breeds and Holstein cows.

Table 1 presents the coefficients of variation (CV) for fat content in different milk samples across dairy cows breeds. The CV of the milk fat content was the highest in SX, with the maximum value being 10-fold higher than the minimum. In contrast, the lowest CV was observed for the BH sample, with the maximum value being 3-fold higher than the minimum.

### 2.2. Fatty Acid Content of Milk Samples

The contents of 17 fatty acids in all milk samples are presented in Table 2. The fatty acid content of milk samples differed significantly among breeds. The most abundant fatty acids in the milk of the different breeds were C14:0, C16:0, C18:0, and C18:1, while the least abundant were C20:1, C20:4, C20:5, and C22:6.

Among dairy cows, all fatty acid contents were significantly higher in JS samples, excluding C22:6, than in BH samples. JS had significantly higher C6:0, C8:0, C10:0, C12:0, C14:0, C16:0, C18:0, C18:1, C20:1, and C20:5 contents than HH; however, significant differences were not observed in the other fatty acids. In Holstein milk from different regions (BH and HH), the contents of all fatty acids differed significantly, excluding C8:0, C10:0, C12:0, C14:0, C18:0, and C20:5.

In the milk of dairy/meat cows, XH samples contained significantly higher C18:0, C18:1, C18:3, C20:1, C20:5, and C22:6 contents than other breeds. Additionally, the fatty acid content of SH samples was significantly higher than that of SI and SX samples, excluding C18:0, C18:1, C18:3, C20:1, and C22:6. Meanwhile, only C8:0 content was significantly higher in SI samples than SX samples; significant differences were not observed between SI and SX in any other fatty acids.

Compared with BH milk samples, XH samples contained significantly higher levels of all fatty acids, excluding C6:0, whereas SH contained significantly higher levels of all fatty acids, excluding C6:0, C18:0, C18:2, and C18:3. The C14:1, C18:3, C20:1, C20:5, and C22:6 contents in XH and C14:1 in SH were more than twice as high as in BH. However, the C6:0, C10:0, C12, C14:0, and C18:2 contents in SI were significantly lower than in BH. Similar results were obtained for SX. Additionally, the C22:6 content in SI milk samples was significantly higher than in BH. The C12:0, C14:0, C16:0, C18:0, and C18:2 contents were significantly lower in JS samples than in milk samples of the four dairy/meat cows. Moreover, XH had significantly higher levels of C18:3, C20:5, and C22:6 than JS, whereas C22:6 was higher in SI; the contents of SX samples did not differ significantly from JS samples. Among all milk samples, XH had the highest C20:5 and C22:6 contents.

Table 3 presents the CVs for the fatty acid contents in the different milk samples. The larger CVs among the cow breeds were concentrated in C20:5 and C22:6. The CVs for C16:1 in SX, C20:5 in JS, SI, and SX, and C22:6 in HH, SH, and SX exceeded 90%. SX had the largest CV for C22:6, with the maximum value 149-fold greater than the minimum. JS had the CV for C14:0, with the maximum value 4-fold larger than the minimum. The CVs of JS for all fatty acids were less than those of HH, excluding C14:1 and C20:5. In Holstein milk from different regions, the CVs for all fatty acids were smaller in BH than in HH. The CVs for all fatty acids were larger for SX samples than SH and XH. Additionally, the CVs for all fatty acids in SX samples were larger than for SI samples, excluding C10:0, C18:0, C18:1, and C18:2. The CVs for all fatty acid contents were larger in the milk of the four dairy/meat cows than for BH, indicating greater intraspecific variation than in BH samples.

### 2.3. Fatty Acid Types in Milk Samples

Table 4 presents the different fatty acid types in different milk samples. Cow breed significantly impacted the milk fatty acid composition. All milk samples contained the highest SFA content and lowest PUFA content. Among dairy cows, significant differences were observed in the SFA, MUFA, and PUFA contents in Holstein milk from different regions. JS had significantly higher SFA and MUFA contents than Holstein milk; however, the MUFA content was only significantly higher in JS than in BH, not HH. In dairy cow milk, the SFA, MUFA, and PUFA contents were significantly higher in XH and SH samples than in SI and SX. XH had significantly higher levels of MUFA than SH. No significant differences were detected in the fatty acid contents of SI and SX samples.

Compared to BH, XH and SH had significantly higher SFAs, MUFAs, and PUFAs, while SI and SX had significantly lower SFAs. In addition, SI contained significantly lower PUFA levels than BH. The contents of all fatty acid types were significantly lower in the milk of the three dairy/meat cows, excluding XH, than in JS; XH exhibited significantly lower SFA levels than JS. Additionally, SI and SX samples had significantly lower SFA levels than those from the three dairy cows. The PUFA content of SI was also significantly lower than samples from the three dairy cows.

In the milk of dairy cows, significant differences were detected in the n-3 and n-6 PUFA contents in Holstein milk from different regions in China. The n-3 and n-6 PUFA contents of JS samples were significantly higher in BH samples but did not differ significantly from HH. In the dairy/meat cows, the n-6 PUFA content was significantly higher in XH and SH than in SI and SX, whereas the n-3 PUFA content was significantly higher in XH than in SH, SI, and SX samples. Additionally, no significant differences were observed in n-6 or n-3 PUFA contents between SI and SX samples.

XH and SH contained significantly higher levels of n-6 PUFAs than BH. In addition, the n-3 PUFA content in XH was significantly higher than in BH. The n-6 PUFA content was significantly lower in SI and SX than in BH, while the n-3 PUFA content in SI and SX did not differ from BH. The n-6 and n-3 PUFA contents of all dairy/meat cow milk were significantly lower than in JS samples, excluding the n-3 PUFA content in XH.

### 2.4. Proportions of Different Types of Fatty Acids in Milk Samples

**Figure 2:**
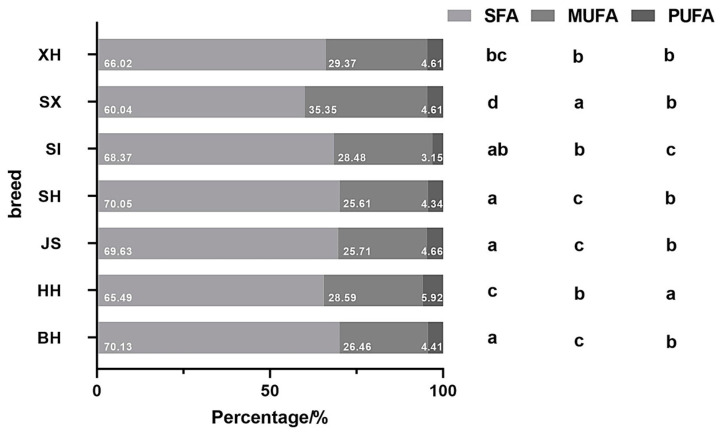
Proportions of fatty acid types in milk samples based on cow breed and region. BH (*n* = 115), HH (*n* = 348), JS (*n* = 94), SH (*n* = 84), SI (*n* = 82), SX (*n* = 38), XH (*n* = 78). Values with different letters differ significantly at *p* < 0.05. BH = Beijing Holstein milk, HH = Hebei Holstein milk, JS = Jersey milk, MUFA = monounsaturated fatty acid, PUFA = polyunsaturated fatty acids, SFA = saturated fatty acid, SH = Sanhe milk, SI = Simmental milk, SX = Shu Xuanhua milk, XH = Xinjiang brown milk.

Among all samples, the SFAs comprised the highest proportion, and PUFAs accounted for the lowest proportion of all fatty acids (Figure 2). Among dairy cow milk samples, the proportions of SFAs, MUFAs, and PUFAs in JS differed significantly from HH but not BH. Significant differences were also observed in the proportion of SFAs, MUFAs, and PUFAs in Holstein milk samples from the different regions. In dairy cow milk, SX had the lowest percentage of SFAs and the highest percentage of MUFAs. SI had the lowest PUFA proportion among all samples, significantly lower than the other three dairy cow milk samples.

Compared with dairy cow milk, SX and XH had significantly lower proportions of SFAs than BH and JS. Meanwhile, SI, SX, and XH contained significantly higher proportions of MUFAs than BH and JS. The proportion of PUFAs in the milk of dairy/meat cows did not differ significantly between BH and JS but was significantly lower in SI. Overall, neither the SFA nor PUFA proportions of dairy/meat cow milk were higher than that of BH, and the PUFA proportion was not higher than any of the three dairy cow milk samples.

**Figure 3:**
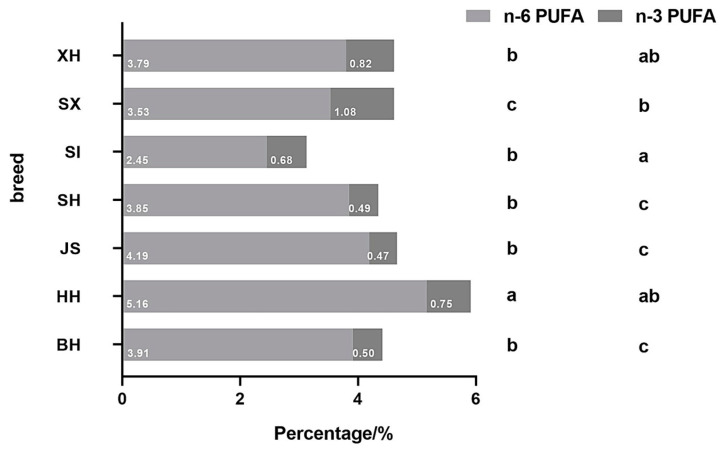
Proportion of different polyunsaturated fatty acid types to total fatty acids in milk of different cow breeds and regions in China. BH (*n* = 115), HH (*n* = 348), JS (*n* = 94), SH (*n* = 84), SI (*n* = 82), SX (*n* = 38), XH (*n* = 78). Values with different letters differ significantly at *p* < 0.05. BH = Beijing Holstein milk, HH = Hebei Holstein milk, JS = Jersey milk, PUFA = polyunsaturated fatty acids, SH = Sanhe milk, SI = Simmental milk, SX = Shu Xuanhua milk, XH = Xinjiang brown milk.

Figure 3 presents the ratios of different PUFA types to total fatty acids in milk samples. HH and SI had the highest (5.17%) and lowest (2.47%) n-6 PUFA proportions, respectively. No significant differences were observed in the proportion of n-6 PUFAs of BH, JS, SH, XH, and SX samples. SX had the highest proportion of n-3 PUFAs (1.08%), significantly higher than in BH, JS, and SH samples. JS had the lowest n-3 PUFA proportion (0.48%), significantly lower than in HH, XH, SI, and SX samples. Additionally, HH had a significantly higher percentage of n-6 and n-3 PUFAs than BH. Overall, milk from dairy cows had a lower n-6 PUFA proportion than dairy cows; however, the n-3 PUFA proportion was higher than in BH.

### 2.5. Protein Content in Milk Samples

**Figure 4:**
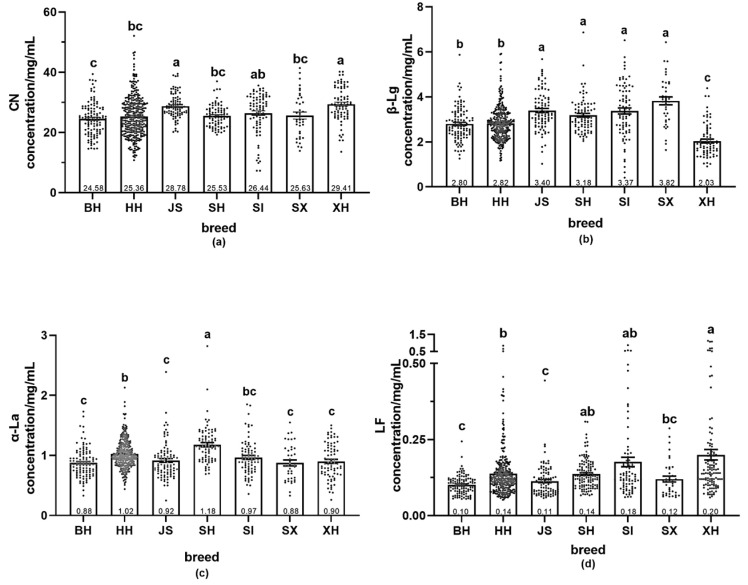
Protein content (mg/mL) in the milk of different cow breeds. (**a**) CN content; (**b**) β-Lg content; (**c**) α-La content; (**d**) LF content. BH (*n* = 112), HH (*n* = 341), JS (*n* = 93), SH (*n* = 84), SI (*n* = 82), SX (*n* = 38), XH (*n* = 78). Values with different letters differ significantly at *p* < 0.05. BH = Beijing Holstein milk, CN = casein, HH = Hebei Holstein milk, JS = Jersey milk, LF = lactoferrin, SH = Sanhe milk, SI = Simmental milk, SX = Shu Xuanhua milk, XH = Xinjiang brown milk, α-La = α-lactalbumin, β-Lg = β-lactoglobulin. Each point indicates a different milk protein concentration for each cow.

The contents of different proteins differed significantly in milk among breeds (Figure 4). In all milk samples, CN was the most abundant protein. In whey protein, the most abundant fraction was β-β-Lg and the least abundant was LF.

XH had the highest CN content (29.41 mg/mL), significantly higher than Holstein milk, and the lowest β-Lg content (2.03 mg/mL), significantly lower than the other breeds. In addition, XH had the highest LF content (0.20 mg/mL), significantly higher than the milk of the three dairy cows and twice as high as in BH. The highest β-Lg content was detected in SX samples (3.82 mg/mL), significantly higher than in Holstein milk. SH had the highest α-La content (1.18 mg/mL), significantly higher than other breeds. In Holstein milk from different regions, the content of α-La and LF was significantly higher in HH than in BH. Additionally, CN and β-Lg contents were significantly higher in JS than in Holstein milk, while α-La and LF contents were significantly higher in HH than in BH and JS. No significant differences were detected in the contents of the four proteins in SH, SI, or SX samples. Overall, the LF content in dairy cow milk was higher than in dairy cow milk.

Table 5 presents the CVs of protein content in the different milk samples. LF had the largest CV among all proteins. XH had the largest LF CV, with the maximum value 17-fold the minimum value, suggesting that the LF content of XH varied among individual animals. The CVs for all four proteins of SI and SX were larger than those of BH, indicating that the protein contents of SI and SX were more strongly affected by individual differences. JS samples had smaller CVs for the more abundant proteins (CN and β-Lg) than Holstein milk; however, larger CVs were observed for the less abundant proteins (α-La and LF) than in Holstein milk.

### 2.6. Proportions of Different Protein Types Among Milk Samples

**Figure 5:**
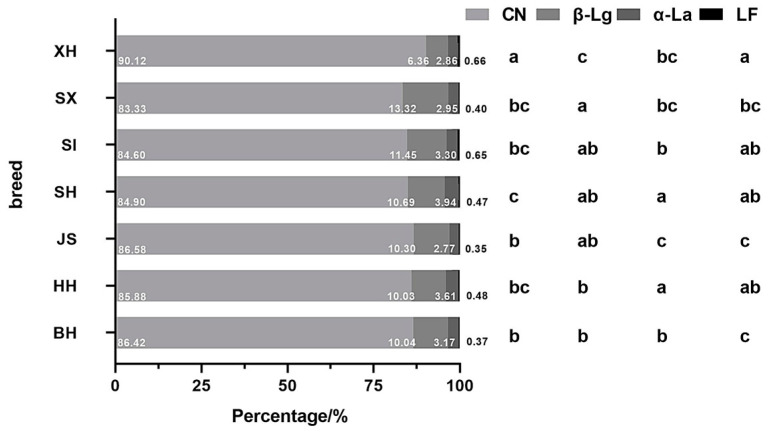
Proportions of different protein types to total proteins in the milk of different cow breeds and regions in China. BH (*n* = 112), HH (*n* = 341), JS (*n* = 93), SH (*n* = 84), SI (*n* = 82), SX (*n* = 38), XH (*n* = 78). Values with different letters differ significantly at *p* < 0.05. BH = Beijing Holstein milk, CN = casein, HH = Hebei Holstein milk, JS = Jersey milk, LF = lactoferrin, SH = Sanhe milk, SI = Simmental milk, SX = Shu Xuanhua milk, XH = Xinjiang brown milk, α-La = α-lactalbumin, β-Lg = β-lactoglobulin.

Figure 5 presents the proportions of different protein types to total proteins in milk samples. JS had the lowest proportion of α-La, significantly lower than in Holstein milk. The lowest LF content was observed in JS and was significantly lower than in HH. In Holstein milk from different regions, the proportions of α-La and LF were significantly higher in HH than BH. XH contained the highest proportion of CN and the lowest proportion of β-Lg, significantly higher and lower than the milk of other breeds, respectively. No significant differences were observed in the proportion of the four proteins between the SI and SX samples.

Compared to BH, SH and XH had a significantly higher proportion of LF, and SI and SX had a significantly higher percentage of β-Lg. SH and SI had significantly higher proportions of α-La and LF than JS.

## 3. Discussion

The results of the current study revealed a significant effect of cow breed on the fat, fatty acid, and protein contents of milk. The geographical region also significantly impacted the fatty acid content, and α-La and LF protein contents, with HH was higher than BH.

Milk fat is a high-value component that provides numerous fat-soluble vitamins. It has a digestibility of more than 98% in the human gastrointestinal tract and is an important indicator of milk quality [20]. The mean fat content of the milk of all dairy cows was higher than that of the dairy/meat cows, with JS milk having the highest mean fat content. This is consistent with Lim et al. [21] and Yamada et al. [22], who also reported higher fat content in JS milk samples than in Holstein samples. The mean fat content of XH was second only to that of JS but varied widely among individuals, with the milk from one individual containing 109.3 mg/mL fat. Meanwhile, geographic region impacted milk fat content less than breed. HH milk samples exhibited greater individual variation in fat content than BH samples, with one individual reaching 108.4 mg/mL. These individual differences may be due to different lactation periods, which can affect milk fat content [23].

Wang et al. [24] reported that SFAs are the most abundant type of fatty acids in milk, followed by MUFAs and PUFAs, respectively. This corresponded with the results of our study. The most abundant SFA among all samples was C16:0; JS samples contained the highest C16:0 content, which align with previous findings [25]. In contrast to the fat content results, the highest mean content of C18:1 was in XH, not JS samples. Oleic acid (C18:1), the most abundant MUFA in milk, has beneficial anti-inflammatory effects in autoimmune diseases, protective effects against cancer, and improves immune system function [26].

PUFAs can also regulate a wide range of homeostatic and inflammatory processes associated with various diseases, either directly or through conversion into locally acting bioactive metabolites. Among these, n-6 and n-3 PUFAs are essential fatty acids that cannot be synthesized by the human body; hence, they can only be obtained from external sources. These fatty acids are essential for brain and vision development, act as natural lipid-lowering agents, reduce the risk of cardiovascular syndromes, and may prevent the progression of insulin resistance and obesity [27,28]. In the current study, the JS milk samples contained the highest mean PUFA contents among all samples. In contrast, Wei et al. [29] found that the PUFA content of JS from Jiangsu Province, China, was lower than that of Chinese Holstein milk. This discrepancy may be related to the region, influenced by geographical location and climatic conditions, etc. In addition, the n-6 to n-3 PUFA ratio is recognized as an important determinant of health; an increased ratio is highly pro-thrombotic and pro-inflammatory and contributes to the prevalence of atherosclerosis, obesity, diabetes, and inflammatory autoimmune diseases. Although the optimal ratio may vary depending on the disease, generally, a lower n-6 to n-3 PUFA ratio is favorable for reducing the risk of many chronic diseases highly prevalent in Western societies and developing countries [30]. Interestingly, JS milk samples had the highest n-6 to n-3 PUFA ratio. In contrast, SI had the lowest n-6 to n-3 PUFA ratio and may be more beneficial for human health.

The dietary levels of milk proteins play a key role in infant and adult health. CN is the main protein in cow milk, comprising ~80% of the total protein and 20% of whey protein. Meanwhile, β-Lg represents approximately 50% of whey protein and can bind to fatty acids and stimulate lipase activity [31]; however, as it is not present in human milk, β-Lg is the main allergen in infants [32]. In contrast, α-La is the major protein in human milk, accounting for ~22% of bovine whey protein; it binds metal ions and participates in lactose synthesis [33]. LF is a functional protein in milk with various beneficial effects, including antibacterial, antiviral, antifungal, anti-inflammatory, and anticancer properties [34]. Although human milk is ideal for newborns, breastfeeding is not always possible. In these instances, infant formula serves as the primary food source for newborns. These formulas have been developed as substitutes for breast milk in terms of nutrients. However, the proteins in infant formulas are primarily derived from cow milk or bovine whey powder. Therefore, it is essential to identify cow milk comparable to human milk. In this study, the low β-Lg content of XH samples was accompanied by a high LF content, which is beneficial to infant health. In fact, among all samples, the milk from one XH cow contained the highest LF level (1.13 mg/mL), similar to the LF level in normal human milk [35]. Additionally, individuals from the HH, XH, and SI groups produced milk with LF levels > 0.5 mg/mL. This variation may be due to differences in lactation. Jingting et al. [36] reported that the LF content of Chinese Holstein milk from Beijing was 0.0949 mg/mL, similar to that of BH but lower than the other breeds in the current study. Moreover, SH milk samples contained the highest average α-La content (1.18 mg/mL), higher than that reported (0.76 mg/mL) in a previous study [37]. In contrast to the current study results, others have reported an average CN content in milk of 32.66 g/L, average β-Lg content of 5.98 g/L, and average α-La content of 1.01 g/L [38]. The primary cause of these differences likely involves inter-country differences, indirectly verifying the influence of the external environment on milk proteins.

## 4. Materials and Methods

### 4.1. Milk Collection

Milk samples were collected in China from September 2022 through February 2023. BH samples were collected from Beijing, HH from Hebei Province, JS from Hebei Province, SH from the Inner Mongolia Autonomous Region, XH and SI from the Xinjiang Uygur Autonomous Region, and SX from Sichuan Province. Cows were fed total mixed ration (TMR) according to national standards, and were adult cows in early to late lactation, with litter sizes ranging from 1–10. BH and HH cows were sampled in the same month, while the other breeds were sampled once per month. A total of 839 milk samples from seven herds were collected: BH (*n* = 115), HH (*n* = 348), JS (*n* = 94), SH (*n* = 84), SI (*n* = 82), SX (*n* = 38), and XH (*n* = 78). All samples were collected via machine milking, immediately placed on dry ice, and analyzed within one month.

### 4.2. Reagents

Seventeen types of fatty acid methyl ester standards were used: methyl caproate, methyl caprylate, methyl caprate, methyl laurate, methyl myristate, methyl pentadecanoate, methyl palmitate, methyl stearate, methyl cis-9-myristoleate, methyl cis-9-palmitoleate, methyl cis-9-oleate, methyl cis-9-12-linoleate, methyl cis-9-12-15-linolenate, methyl cis-11-eicosenoic, methyl cis-5-8-11-14-arachidonate, methyl cis-4-7-10-13-16-19-eicosapentaenoate, and methyl cis-5-8-11-14-docosahexaenoic acid (J&K Scientific, Beijing, China). Three types of whey protein standards were used: β-lactoglobulin, α-lactalbumin, and lactoferrin (Sigma-Aldrich, Beijing, China). Other reagents included anhydrous ethanol, ammonia (25–28%), petroleum ether (boiling range 30–60 °C), ethyl ether, sodium hydroxide, anhydrous sodium sulfate, sodium chloride, boron trifluoride methanol solution (10%), methanol, n-heptane, ultrapure water, hydrochloric acid, acetonitrile, and trifluoroacetic acid (Macklin, Shanghai, China).

### 4.3. Milk Fat Measurement

Milk fat was determined using the third method, alkaline hydrolysis, of the “GB 5009.6-2016 National Standard for Food Safety—Determination of Fat in Foods” [39] with slight modifications. In brief, frozen cow milk was thawed in a 4 °C refrigerator and vortexed. Next, 1 mL of milk was thoroughly mixed with 1 mL of anhydrous ethanol and 1 mL of ammonia. The solution was placed in a water bath at 55 °C for 20 min for hydrolysis. Subsequently, the samples were cooled to room temperature. Next, 3 mL of ethanol was mixed thoroughly with the hydrolyzed milk, 5 mL of ether was added, and shaken for 1 min. Then, 5 mL of petroleum ether was added and shaken for 30 s. The solution was thoroughly mixed to extract the fat, allowed to stand for 30 min to separate the layers, and the upper layer of the solution was collected. The extraction process was repeated, the extracts were combined, and the mixture was placed in a boiling water bath to evaporate the solvent, creating a milk fat residue. The samples were dried to constant weight, and the fat content was recorded.

### 4.4. Milk Fatty Acid Measurement

Milk fatty acid was determined using the second external standard method of the “GB 5009.168-2016 National Standard for Food Safety—Determination of Fatty Acids in Foods” [40], with slight modifications.

#### 4.4.1. Methylation of Milk Fatty Acids

To the extracted milk fat, 10 mL of 2% sodium hydroxide methanol solution was added and heated in a metal bath at 81 °C until the oil droplets disappeared. Next, 7 mL of a 10% boron trifluoride methanol solution was added and heated for 2 min for methyl esterification. After heating, 10 mL of n-heptane and 5 mL of saturated sodium chloride solution were added, thoroughly mixed, shaken, and left to stratify. Next, 3 mL of the extract was added to 2 g of anhydrous sodium sulfate to remove the water. The layers were then allowed to separate, and the upper layer of the clarified solution was collected and filtered.

#### 4.4.2. Milk Fatty Acid Methyl Esters Measurement

Fatty acid methyl esters were used for gas chromatograph detection of 17 fatty acids: caproic acid (C6:0), octanoic acid (C8:0), decanoic acid (C10:0), lauric acid (C12:0), myristic acid (C14:0), pentadecanoic acid (C15:0), palmitic acid (C16:0), stearic acid (C18:0), myristoleic acid (C14:1), palmitoleic acid (C16:1), oleic acid (C18:1), linoleic acid (C18:2), linolenic acid (C18:3), eicosenoic acid (C20:1), arachidonic acid (C20:4), eicosapentaenoic acid (C20:5), and docosahexaenoic acid (C22:6). The gas chromatograph was equipped with a hydrogen flame ionization detector. The capillary column model was a DB-WAX (30 m × 0.25 mm × 0.25 μm). The temperature of the inlet was 250 °C, and the temperature of the detector was 280 °C. Nitrogen was used as a carrier gas at a flow rate of 1 mL/min. The injection volume was 1 μL. The initial temperature was 50 °C for 1 min, which then increased at 25 °C/min to 200 °C, and then at 3 °C/min to 230 °C for 10 min. Different concentrations of fatty acid standards were prepared using n-heptane and detected under the same chromatographic conditions. A standard curve was established with the concentration as the horizontal coordinate and the peak area as the vertical coordinate. The external standard method was used to characterize the standard based on its retention time and quantify the peak area.

### 4.5. Milk Protein Measurement

#### 4.5.1. Extraction of Milk Proteins

Frozen buttermilk was thawed in a 4 °C refrigerator and vortexed. Next, 5 mL of milk was adjusted to pH 4.60 ± 0.01 using 1 mol/L HCL. After precipitation at room temperature for 1 h, the milk was centrifuged at 4 °C and 15,000 rpm for 30 min. The upper fat layer was removed, and the milk was centrifuged under the same conditions.

#### 4.5.2. Casein Content Measurement

The method of casein (CN) determination was based on that of Han et al. [41], with minor modifications. The bottom layer of the precipitate was washed sequentially with 1 mL of distilled water, followed by ethanol, an ethanol–ether 1:1 mixture, and finally with ether. It was dried at 60 °C in a drying oven for 2 h and cooled in a glass desiccator for 30 min to obtain CN.

#### 4.5.3. Whey Protein Measurement

The whey protein determination method was used per Ostertag et al. [42] with minor modifications. A volume of 100 μL of the upper layer of the clear solution was diluted ten-fold, filtered, and subjected to high-performance liquid chromatography to detect three kinds of whey proteins: β-lactoglobulin (β-Lg), α-lactalbumin (α-La), and lactoferrin (LF).

High-performance liquid chromatography was performed with a UV detector and a ZORBAX SB 300 C8 (Agilent Technologies, Beijing, China) column (4.6 mm × 250 mm × 5 µm). The following conditions were applied: column oven temperature of 45 °C, detection wavelength of 210 nm, mobile phase A of 0.1% trifluoroacetic acid-water solution, mobile phase B of 0.1% trifluoroacetic acid-acetonitrile solution, flow rate of 0.8 mL/min, and injection volume of 20 μL. Gradient elution was performed with an initial mobile phase B ratio of 35% for 1 min, increased to 60% at 8 min, and increased to 70% at 9 min for 1 min. Whey protein standards of different concentrations were prepared in ultrapure water and analyzed under the same chromatographic conditions. The standard curve was established with the concentration as the horizontal coordinate and the peak area as the vertical coordinate. The external standard method was used to characterize the standard based on its retention time and quantify the peak area.

### 4.6. Statistical Analyses

Microsoft Excel was used to process the data; the results were expressed as mean ± standard error of the mean (Mean ± SEM). The Kruskal–Wallis test in SPSS statistical 27 software was used to analyze the data for significance. Statistical significance was set at *p* < 0.05. The coefficient of variation (%) was determined as the ratio of the standard deviation and mean × 100.

## 5. Conclusions

JS samples contained the highest mean fat, SFA, MUFA, PUFA, and β-Lg contents, similar to other studies [24,43]. HH samples had a lower average PUFA content than JS and the highest proportion of PUFAs, which may be of concern. Chinese Holstein cows and JS are the major dairy cows in China, while SH, SI, SX, and XH are the main dairy/meat cattle. We found that SH, SI, and SX had higher levels of LF compared to those in other milk samples. Therefore, it may be possible to use this to increase the development of dairy/meat milk in the future. In addition, XH samples also had the lowest mean β-Lg content, which sensitizes infants, and the highest mean LF content—the functionally active substance. XH had significantly higher SFA, MUFA, and PUFA content than BH. Hence, XH milk has a more favorable protein composition and PUFA content for infant health than JS.

In conclusion, origin in China did not significantly impact total fat and major protein (CN and β-Lg) contents in milk but did affect fatty acid (C16:0 and C18:1), α-La, and LF contents in Chinese Holstein cows. The breed also significantly affected fatty acid and protein contents and ratios. Individuals producing high functional fatty acids and protein contents are of particular interest and may be further examined for better exploitation in the future. In addition, the impact of other influencing factors, such as lactation period, individual age of the litter, health status, etc., should be evaluated in the future to provide reference values for improving the quality and nutritional value of cow milk.

## Figures and Tables

**Table 1:** Coefficient of variation of fat content in milk from different cow breeds and regions in China.

Breed	Coefficient of Variation (%)
BH (*n* = 115)	19.19
HH (*n* = 348)	33.73
JS (*n* = 94)	21.11
SH (*n* = 84)	41.79
SI (*n* = 82)	49.37
SX (*n* = 38)	53.28
XH (*n* = 78)	42.41

BH = Beijing Holstein milk, HH = Hebei Holstein milk, JS = Jersey milk, SH = Sanhe milk, SI = Simmental milk, SX = Shu Xuanhua milk, XH = Xinjiang brown milk.

**Table 2:** Fatty acid content (mg/mL) of milk samples from different cow breeds and regions in China.

Fatty Acid	Breed						
	BH (*n* = 115)	HH (*n* = 348)	JS (*n* = 94)	SH (*n* = 84)	SI (*n* = 82)	SX (*n* = 38)	XH (*n* = 78)
Caproic acid C6:0	0.467 ± 0.013 ^b^	0.384 ± 0.010 ^c^	0.590 ± 0.016 ^a^	0.553 ± 0.026 ^ab^	0.236 ± 0.015 ^d^	0.143 ± 0.014 ^d^	0.555 ± 0.030 ^ab^
Octanoic acid C8:0	0.410 ± 0.014 ^c^	0.422 ± 0.010 ^c^	0.668 ± 0.019 ^a^	0.610 ± 0.028 ^ab^	0.340 ± 0.019 ^c^	0.154 ± 0.013 ^d^	0.634 ± 0.036 ^a^
Decanoic acid C10:0	0.686 ± 0.025 ^c^	0.640 ± 0.017 ^c^	1.190 ± 0.045 ^a^	0.942 ± 0.046 ^b^	0.417 ± 0.027 ^d^	0.210 ± 0.020 ^d^	0.995 ± 0.060 ^ab^
Lauric acid C12:0	0.902 ± 0.029 ^c^	0.877 ± 0.026 ^c^	1.699 ± 0.069 ^a^	1.268 ± 0.066 ^b^	0.553 ± 0.037 ^d^	0.293 ± 0.028 ^d^	1.340 ± 0.087 ^b^
Myristic acid C14:0	2.392 ± 0.063 ^c^	2.739 ± 0.078 ^c^	4.437 ± 0.109 ^a^	3.369 ± 0.148 ^b^	1.760 ± 0.113 ^d^	1.072 ± 0.113 ^d^	3.720 ± 0.218 ^b^
Myristoleic acid C14:1	0.196 ± 0.006 ^b^	0.396 ± 0.010 ^a^	0.431 ± 0.024 ^a^	0.468 ± 0.023 ^a^	0.159 ± 0.011 ^b^	0.120 ± 0.015 ^b^	0.459 ± 0.032 ^a^
Pentadecanoic acid C15:0	0.194 ± 0.006 ^b^	0.275 ± 0.010 ^a^	0.335 ± 0.014 ^a^	0.357 ± 0.018 ^a^	0.189 ± 0.013 ^b^	0.158 ± 0.022 ^b^	0.309 ± 0.019 ^a^
Palmitic acid C16:0	7.120 ± 0.194 ^c^	9.786 ± 0.242 ^b^	10.913 ± 0.281 ^a^	9.277 ± 0.429 ^b^	5.758 ± 0.365 ^cd^	3.971 ± 0.397 ^d^	9.547 ± 0.503 ^b^
Palmitoleic acid C16:1	0.329 ± 0.010 ^b^	0.630 ± 0.017 ^a^	0.518 ± 0.017 ^a^	0.536 ± 0.028 ^a^	0.261 ± 0.017 ^b^	0.302 ± 0.046 ^b^	0.537 ± 0.031 ^a^
Stearic acid C18:0	1.995 ± 0.057 ^cd^	2.402 ± 0.081 ^c^	3.869 ± 0.126 ^a^	2.119 ± 0.110 ^cd^	1.744 ± 0.136 ^d^	1.467 ± 0.127 ^d^	3.208 ± 0.169 ^b^
Oleic acid C18:1	4.571 ± 0.127 ^c^	6.510 ± 0.169 ^b^	7.742 ± 0.242 ^a^	5.821 ± 0.348 ^b^	4.140 ± 0.283 ^bc^	4.046 ± 0.392 ^c^	7.934 ± 0.458 ^a^
Linoleic acid C18:2	0.738 ± 0.020 ^c^	1.298 ± 0.031 ^a^	1.356 ± 0.051 ^a^	0.923 ± 0.039 ^bc^	0.345 ± 0.022 ^d^	0.379 ± 0.033 ^d^	1.055 ± 0.051 ^b^
Linolenic acid C18:3	0.094 ± 0.002 ^c^	0.194 ± 0.006 ^ab^	0.148 ± 0.005 ^b^	0.116 ± 0.005 ^c^	0.099 ± 0.007 ^c^	0.124 ± 0.016 ^c^	0.231 ± 0.015 ^a^
Eicosenoic acid C20:1	0.044 ± 0.001 ^c^	0.071 ± 0.002 ^b^	0.086 ± 0.004 ^a^	0.068 ± 0.004 ^b^	0.052 ± 0.005 ^c^	0.061 ± 0.009 ^bc^	0.091 ± 0.005 ^a^
Arachidonic acid C20:4	0.0458 ± 0.0018 ^b^	0.0710 ± 0.0020 ^a^	0.0821 ± 0.004 ^a^	0.0712 ± 0.0039 ^a^	0.0308 ± 0.0029 ^c^	0.0411 ± 0.0058 ^bc^	0.0820 ± 0.0046 ^a^
Eicosapentaenoic acid C20:5	0.0040 ± 0.0002 ^c^	0.0047 ± 0.0002 ^c^	0.0085 ± 0.0008 ^b^	0.0060 ± 0.0005 ^b^	0.0022 ± 0.0003 ^d^	0.0044 ± 0.0010 ^cd^	0.0121 ± 0.0009 ^a^
Docosahexaenoic acid C22:6	0.0019 ± 0.0001 ^d^	0.0030 ± 0.0002 ^bc^	0.0028 ± 0.0002 ^bcd^	0.0034 ± 0.0003 ^b^	0.0030 ± 0.0002 ^bc^	0.0028 ± 0.0007 ^cd^	0.0047 ± 0.0003 ^a^

Values with different letters are significantly different at *p* < 0.05. BH = Beijing Holstein milk, HH = Hebei Holstein milk, JS = Jersey milk, SH = Sanhe milk, SI = Simmental milk, SX = Shu Xuanhua milk, XH = Xinjiang brown milk. Results are presented as mean ± standard error of the mean (SEM).

**Table 3:** Fatty acid content coefficients of variation (%) in milk samples from different cow breeds and regions in China.

Fatty Acid	Breed						
	BH (*n* = 115)	HH (*n* = 348)	JS (*n* = 94)	SH (*n* = 84)	SI (*n* = 82)	SX (*n* = 38)	XH (*n* = 78)
C6:0	30.80	47.06	26.19	43.08	57.41	59.67	48.11
C8:0	37.92	44.30	28.11	41.73	51.80	53.48	49.88
C10:0	38.34	48.69	37.06	45.14	59.31	57.81	53.68
C12:0	34.46	54.81	39.50	47.53	60.10	60.01	57.31
C14:0	28.47	53.39	23.72	40.26	58.02	64.80	51.80
C14:1	34.53	47.37	54.34	45.11	64.21	76.27	60.78
C15:0	32.75	66.13	41.54	47.54	63.65	86.47	53.31
C16:0	29.25	46.05	24.94	42.36	57.35	61.68	46.55
C16:1	33.91	51.78	32.58	47.40	59.17	93.26	51.47
C18:0	30.57	63.14	31.55	47.79	70.59	53.29	46.55
C18:1	29.86	48.31	30.29	54.72	61.94	59.69	51.03
C18:2	28.36	45.02	36.12	38.67	56.71	52.89	42.50
C18:3	26.08	57.98	29.82	41.24	59.58	79.85	57.38
C20:1	30.96	58.60	45.64	53.66	84.96	93.24	49.59
C20:4	42.68	52.85	47.01	50.58	84.69	87.75	49.81
C20:5	58.94	73.95	96.20	74.30	109.70	144.81	62.17
C22:6	53.67	95.30	75.64	90.62	69.73	158.64	54.37

BH = Beijing Holstein milk, HH = Hebei Holstein milk, JS = Jersey milk, SH = Sanhe milk, SI = Simmental milk, SX = Shu Xuanhua milk, XH = Xinjiang brown milk.

**Table 4:** Different types of fatty acids (mg/mL) in milk samples of cows of different breeds and regions in China.

Fatty Acid	Breed						
	BH (*n* = 115)	HH (*n* = 348)	JS (*n* = 94)	SH (*n* = 84)	SI (*n* = 82)	SX (*n* = 38)	XH (*n* = 78)
SFA	14.165 ± 0.354 ^c^	17.523 ± 0.435 ^b^	23.701 ± 0.552 ^a^	18.494 ± 0.819 ^b^	10.997 ± 0.699 ^d^	7.467 ± 0.698 ^d^	20.308 ± 1.074 ^b^
MUFA	5.139 ± 0.139 ^d^	7.607 ± 0.191 ^b^	8.777 ± 0.269 ^a^	6.893 ± 0.385 ^c^	4.612 ± 0.311 ^d^	4.530 ± 0.438 ^d^	9.022 ± 0.517 ^ab^
PUFA	0.884 ± 0.023 ^c^	1.571 ± 0.038 ^a^	1.598 ± 0.056 ^a^	1.119 ± 0.046 ^b^	0.480 ± 0.030 ^d^	0.551 ± 0.051 ^cd^	1.384 ± 0.067 ^ab^
n-6 PUFA	0.784 ± 0.021 ^d^	1.369 ± 0.033 ^ab^	1.438 ± 0.053 ^a^	0.994 ± 0.042 ^c^	0.376 ± 0.024 ^e^	0.420 ± 0.037 ^e^	1.137 ± 0.055 ^bc^
n-3 PUFA	0.100 ± 0.002 ^c^	0.201 ± 0.006 ^b^	0.160 ± 0.005 ^b^	0.125 ± 0.006 ^c^	0.105 ± 0.007 ^c^	0.131 ± 0.016 ^c^	0.247 ± 0.016 ^a^
n-6/n-3	7.8	7.5	9.4	8.2	3.8	4.1	5.0

SFA: C6:0, C8:0, C10:0, C12:0, C14:0, C15:0, C16:0, C18:0; MUFA: C14:1, C16:1, C18:1, C20:1; PUFA: C18:2, C18:3, C20:4, C20:5, C22:6 (n-6 PUFA: C18:2, C20:4; n-3 PUFA: C18:3, C20:5, C22:6). Values with different letters are significantly different at *p* < 0.05. BH = Beijing Holstein milk, HH = Hebei Holstein milk, JS = Jersey milk, MUFA, monounsaturated fatty acid, PUFA, polyunsaturated fatty acids, SFA = saturated fatty acid, SH = Sanhe milk, SI = Simmental milk, SX = Shu Xuanhua milk, XH = Xinjiang brown milk.

**Table 5:** Coefficient of variations (%) for the protein content of milk samples.

Protein	Milk Type						
	BH (*n* = 112)	HH (*n* = 341)	JS (*n* = 93)	SH (*n* = 84)	SI (*n* = 82)	SX (*n* = 38)	XH (*n* = 78)
CN	21.98	26.08	13.91	14.17	25.59	27.86	19.42
β-Lg	26.13	25.66	25.22	23.91	35.23	27.56	36.82
α-La	27.18	22.39	32.96	26.03	29.59	34.44	32.05
LF	29.92	59.80	46.55	36.30	79.02	48.07	91.80

BH = Beijing Holstein milk, CN = casein, HH = Hebei Holstein milk, JS = Jersey milk, LF = lactoferrin, SH = Sanhe milk, SI = Simmental milk, SX = Shu Xuanhua milk, XH = Xinjiang brown milk, α-La = α-lactalbumin, β-Lg = β-lactoglobulin.

## Data Availability

The original contributions presented in the study are included in the article/Supplementary Material, further inquiries can be directed to the corresponding authors.

## Supplementary Materials

The following supporting information can be downloaded at: https://www.mdpi.com/article/10.3390/molecules29215142/s1, Table S1: Fat content of milk samples; Table S2: Fat acid content of milk samples; Table S3: content of milk samples.

## References

[1] Beckett E.L., Cassettari T., Starck C., Fayet-Moore F. (2024). Dairy milk: There are alternatives but no equivalents. Food Sci. Nutr..

[2] Park Y.W., Haenlein G.F.W., Wendorff W.L. (2017). Overview of Milk of Non-Bovine Mammals (Second Edition). Handbook of Milk of Non-Bovine Mammals.

[3] Lin T.T., Meletharayil G., Kapoor R., Abbaspourrad A. (2021). Bioactives in bovine milk: Chemistry, technology, and applications. Nutr. Rev..

[4] Tzompa-Sosa D.A., Meurs P.P., van Valenberg H.J.F. (2018). Triacylglycerol Profile of Summer and Winter Bovine Milk Fat and the Feasibility of Triacylglycerol Fragmentation. Eur. J. Lipid Sci. Technol..

[5] Tvrzicka E., Kremmyda L.S., Stankova B., Zak A. (2011). Fatty acids as biocompounds: Their role in human metabolism, health and disease—A review. Part 1: Classification, dietary sources and biological functions. Biomed. Pap. Med. Fac. Univ. Palacky Olomouc. Czech Repub..

[6] Khastayeva A.Z., Zhamurova V.S., Mamayeva L.A., Kozhabergenov A.T., Karimov N.Z., Muratbekova K.M. (2021). Qualitative indicators of milk of Simmental and Holstein cows in different seasons of lactation. Vet. World.

[7] Rezagholivand A., Nikkhah A., Khabbazan M.H., Mokhtarzadeh S., Dehghan M., Mokhtabad Y., Sadighi F., Safari F., Rajaee A. (2021). Feedlot performance, carcass characteristics and economic profits in four Holstein-beef crosses compared with pure-bred Holstein cattle. Livest. Sci..

[8] Zhang S.L., Sun D.X. (2021). Past, now, and future of Dairy breeding Industry. China Livest. Ind..

[9] Liu B. (2022). Comparative Analysis of Nutrient Contents, Fatty Acid Content and Composition of Raw Milk from Dairy Cattle of Different Breeds. Anim. Husb. Feed. Sci..

[10] Huson H.J., Sonstegard T.S., Godfrey J., Hambrook D., Wolfe C., Wiggans G., Blackburn H., VanTassell C.P. (2020). A Genetic Investigation of Island Jersey Cattle, the Foundation of the Jersey Breed: Comparing Population Structure and Selection to Guernsey, Holstein, and United States Jersey Cattle. Front. Genet..

[11] Zhang X.Y., Wang W., Cao Z.J., Yang H.J., Wang Y.J., Li S.L. (2023). Effects of altitude on the gut microbiome and metabolomics of Sanhe heifers. Front. Microbiol..

[12] (2011). Workstation, Inner Mongolia Autonomous Region Livestock Improvement. ShanHe Cattle..

[13] Zhen M., Zhibing Y., Fanrong C., Xiangmin Y., Lixing Y., Yang Z. (2024). Progress of selective breeding of Xinjiang brown cattle and their meat type groups. Chin. Bov. Sci..

[14] Tao Z., Jiaqi L., Lei X., Dan W., Menghua Z. (2024). Detection and Population Structure Analysis of Genomic Structural Variation in Xinjiang Brown Cattle Based on Whole Genome Resequencing Data. J. Anim. Husb. Vet. Sci..

[15] Wei C., Luo H.P., Wang Y.C., Huang X.X., Zhang M.H., Zhang X.X., Wang D., Ge J.J., Xu L., Jiang H. (2021). Analyses of the genetic relationships between lactose, somatic cell score, and growth traits in Simmental cattle. Animal.

[16] Fu M.Z., Wang W., Yi J. (2023). New dairy cattle breed Shu Xuanhua Cattle. Rural. Pundit.

[17] Qiao S., Rong T., Huimin L., Jinguo P., Zhaodong S., Faguang Y., Xinrui W., Hong L. (2023). Comparison and correlation analysis of nutrient composition and bacterial diversity of Holstein and Jersey milk under highland feeding conditions. Food Ind. Sci. Technol..

[18] Zitong Y., Wenan Z., Yu Z., Jishuang T., Ziliang S., Liping H. (2023). Comparative study of rumen volatile fatty acids and milk quality of Holstein, Jersey and Holstein-Jerse crossbred cows in China. Chin. J. Anim. Husb..

[19] Xiaoxu S. (2023). Comparison of Nutrient Composition and Bacterial Diversity of Holstein Milk and Jersey Milk Under Plateau Feeding and Correlation Analysis. Master’s Thesis.

[20] Mohan M.S., O’Callaghan T.F., Kelly P., Hogan S.A. (2021). Milk fat: Opportunities, challenges and innovation. Crit. Rev. Food Sci. Nutr..

[21] Lim D.H., Mayakrishnan V., Ki K.S., Kim Y., Kim T.I. (2021). The effect of seasonal thermal stress on milk production and milk compositions of Korean Holstein and Jersey cows. Anim. Biosci..

[22] Yamada K.D.G., dos Santos G.T., Damasceno J.C., de Almeida K.V., Osorio J.A.C., Lourenco J.C.S., Gurgel A.L.C., Dias-Silva T.P., de Araújo M.J., Itavo L.C.V. (2023). Effects of heat-stress-reducing systems on blood constituents, milk production and milk quality of Holstein and Jersey cows and heifers on pasture. Trop. Anim. Health Prod..

[23] Gathinji P.K., Yousofi Z., Akada K., Wali A., Nishino N. (2023). Monitoring the Milk Composition, Milk Microbiota, and Blood Metabolites of Jersey Cows throughout a Lactation Period. Vet. Sci..

[24] Wang F.G., Chen M.Q., Luo R.B., Huang G.X., Wu X.F., Zheng N., Zhang Y.D., Wang J.Q. (2022). Fatty acid profiles of milk from Holstein cows, Jersey cows, buffalos, yaks, humans, goats, camels, and donkeys based on gas chromatography-mass spectrometry. J. Dairy Sci..

[25] Wang X.D., Zhu H.Q., Zhang W.Y., Zhang Y.M., Zhao P., Zhang S.W., Pang X.Y., Vervoort J., Lu J., Lv J.P. (2022). Triglyceride and fatty acid composition of ruminants milk, human milk, and infant formulae. J. Food Compos. Anal..

[26] Sales-Campos H., de Souza P.R., Peghini B.C., da Silva J.S., Cardoso C.R. (2013). An Overview of the Modulatory Effects of Oleic Acid in Health and Disease. Mini-Rev. Med. Chem..

[27] Zárate R., el Jaber-Vazdekis N., Tejera N., Pérez J.A., Rodríguez C. (2017). Significance of long chain polyunsaturated fatty acids in human health. Clin. Transl. Med..

[28] D’Angelo S., Motti M.L., Meccariello R. (2020). ω-3 and ω-6 Polyunsaturated Fatty Acids, Obesity and Cancer. Nutrients.

[29] Wei N., Mengqi W., Shaoqing Z., Yongliang F., Tao W., Huimin Z. (2018). Analysis of Fatty Acid Composition and Determination of Milk Performance of Holstein and Jersey Cows. Chin. Dairy Cows.

[30] Simopoulos A.P. (2002). The importance of the ratio of omega-6/omega-3 essential fatty acids. Biomed. Pharmacother..

[31] Goulding D.A., Fox P.F., O’Mahony J.A. (2020). Milk Proteins: An Overview.

[32] Pastuszka R., Barlowska J., Litwinczuk Z. (2016). Allergenicity of milk of different animal species in relation to human milk. Postep. Hig. I Med. Dosw..

[33] Layman D.K., Lönnerdal B., Fernstrom J.D. (2018). Applications for α-lactalbumin in human nutrition. Nutr. Rev..

[34] Wang B., Timilsena Y.P., Blanch E., Adhikari B. (2019). Lactoferrin: Structure, function, denaturation and digestion. Crit. Rev. Food Sci. Nutr..

[35] Antoshin A.A., Shpichka A.I., Huang G., Chen K., Lu P., Svistunov A.A., Lychagin A.V., Lipina M.M., Sinelnikov M.Y., Reshetov I.V. (2021). Lactoferrin as a regenerative agent: The old-new panacea?. Pharmacol. Res..

[36] Jingting C., Dengpan B., Lu M., Jinhui Y., Fadi L. (2013). Comparative Analysis of Immunoglobulin and Lactoferrin in Different Bovine Milk. North China J. Agric..

[37] Amalfitano N., Stocco G., Maurmayr A., Pegolo S., Cecchinato A., Bittante G. (2020). Quantitative and qualitative detailed milk protein profiles of 6 cattle breeds: Sources of variation and contribution of protein genetic variants. J. Dairy Sci..

[38] Maurmayr A., Pegolo S., Malchiodi F., Bittante G., Cecchinato A. (2018). Milk protein composition in purebred Holsteins and in first/second-generation crossbred cows from Swedish Red, Montbeliarde and Brown Swiss bulls. Animal.

[39] (2016). National Food Safety Standards Determination of Fat in Food.

[40] (2016). National Food Safety Standards Determination of Fatty Acids in Food.

[41] Han Z.Q., Fang J.H. (2010). Technology of Extracting Casein from Milk in Laboratory. Anim. Husb. Feed. Sci..

[42] Ostertag F., Schmidt C.M., Berensmeter S., Hinrichs J. (2021). Development and validation of an RP-HPLC DAD method for the simultaneous quantification of minor and major whey proteins. Food Chem..

[43] Bangani N.M., Muller C.J.C., Dzama K., Cruywagen C.W.C., Nherera-Chokuda F., Imbayarwo-Chikosi V.E. (2023). Estimating milk production and energy-use efficiency of pasture-grazed Holstein and Jersey cows using mathematical models. South Afr. J. Anim. Sci..

